# Different Impact of Beta-Blockers on Long-Term Mortality in Heart Failure Patients with and without Chronic Obstructive Pulmonary Disease

**DOI:** 10.3390/jcm10194378

**Published:** 2021-09-25

**Authors:** Satoshi Higuchi, Takashi Kohno, Shun Kohsaka, Yasuyuki Shiraishi, Makoto Takei, Ayumi Goda, Satoshi Shoji, Yuji Nagatomo, Tsutomu Yoshikawa

**Affiliations:** 1 Department of Emergency and General Medicine, Kyorin University School of Medicine, Tokyo 181-8611, Japan; 2 Department of Cardiovascular Medicine, Kyorin University School of Medicine, Tokyo 181-8611, Japan; ayumix34@yahoo.co.jp; 3 Department of Cardiology, Keio University School of Medicine, Tokyo 160-8582, Japan; sk@keio.jp (S.K.); yasshiraishi@keio.jp (Y.S.); sshoji0116@gmail.com (S.S.); 4 Department of Cardiology, Saiseikai Central Hospital, Tokyo 108-0073, Japan; makoto_tk@hotmail.com; 5 Department of Cardiology, National Defense Medical College, Tokorozawa 359-8513, Japan; y.nagatomo1111@gmail.com; 6 Department of Cardiology, Sakakibara Heart Institute, Tokyo 183-0003, Japan; tyoshi@shi.heart.or.jp

**Keywords:** heart failure, beta-blocker, chronic obstructive pulmonary disease, heart failure with reduced ejection fraction, heart failure with mid-range ejection fraction

## Abstract

The administration of beta-blockers is challenging and their efficacy is unclear in heart failure (HF) patients with chronic obstructive pulmonary disease (COPD). This study aimed to investigate the association of beta-blockers with mortality in such patients. This multicenter observational cohort study included hospitalized HF patients with a left ventricular ejection fraction <50% and evaluated them retrospectively. COPD was diagnosed based on medical records and/or the clinical judgment of each investigator. The study endpoints were two-year all-cause, cardiac, and non-cardiac mortality. This study included 83 patients with COPD and 1760 patients without. Two-year all-cause, cardiac, and non-cardiac mortality were observed in 315 (17%), 149 (8%), and 166 (9%) patients, respectively. Beta-blockers were associated with lower all-cause mortality regardless of COPD (COPD: hazard ratio [HR] 0.39, 95% CI 0.16–0.98, *p* = 0.044; non-COPD: HR 0.62, 95% CI 0.46–0.83, *p* = 0.001). This association in HF patients with COPD persisted after multivariate analysis and inverse probability weighting and was due to lower non-cardiac mortality (HR 0.40, 95% CI 0.14–1.18. *p* = 0.098), not cardiac mortality (HR 0.37, 95% CI 0.07–2.01, *p* = 0.248). Beta-blockers were associated with lower all-cause mortality in HF patients with COPD due to lower non-cardiac mortality. This may reflect selection biases in beta-blocker prescription.

## 1. Introduction

Beta-blockers are a key drug for heart failure (HF) to improve prognosis and quality of life and recent guidelines recommend these agents for HF with reduced ejection fraction [1,2]. However, underuse of these agents has been noted in patients with chronic obstructive pulmonary disease (COPD) [3,4,5]. Numerous observational studies have suggested that beta-blockers are associated with better prognosis in COPD patients with cardiovascular diseases [6,7]. The Global Initiative for Chronic Obstructive Lung Disease (GOLD) clinical guidelines suggest that cardio selective beta-blockers should be preferred over non-cardio selective beta-blockers for patients with COPD [8]. However, a recent randomized controlled trial, BLOCK-COPD, demonstrated that hospitalization for COPD exacerbation was more common in the metoprolol (one of cardio selective beta-blockers) group than in the placebo group among patients with moderate or severe COPD who did not have an established indication for the agent [9]. Furthermore, flow- and volume-related reversibility were observed in 18.4% of COPD patients [10]. Other previous studies have indicated that a certain number of COPD patients overlapped asthma, and its prevalence ranged from 6% to 55% [11]. This heterogeneity in COPD may also affect the efficacy of beta-blockers.

COPD is a common comorbidity of patients with HF, both of which leads to death worldwide [12]. In addition, COPD has been reported to be a worse long-term predictor in the setting of HF [3]. There are also differences in the prevalence of COPD [5,13,14] and sensitivity for beta-blockers [15,16] among different races and regions. Although such diversity may affect the clinical course of HF patients with COPD, there is little evidence in East Asian populations. Furthermore, the significance of beta-blockers in this region has not been sufficiently investigated. The present study aimed to investigate the clinical impact of beta-blockers and predictors for the prescription in HF patients with COPD.

## 2. Materials and Methods

### 2.1. Study Population

The design of the West Tokyo Heart Failure (WET-HF) registry has been previously reported [17]. Briefly, WET-HF is a large, prospective, multicenter registry designed to collect data on the clinical characteristics and outcomes of patients hospitalized for acute HF (AHF). The diagnosis of AHF was determined by cardiologists at each institution. A diagnosis of AHF is defined as rapid-onset HF or a change in the signs and symptoms of HF requiring urgent therapy and hospitalization, based on the Framingham criteria [18]. Consecutive AHF patients across five academic hospitals were registered from 2006 to 2017. This study protocol conforms to the 1975 Declaration of Helsinki [19] and is in line with the Ethical Guidelines for Epidemiological Research established by the Japanese government. The study was approved by the ethics committee at each hospital and registered on the University Medical Information Network (UMIN 000001171). Written or oral informed consent was obtained from each subject before the registration. All analyses in the current study were conducted retrospectively.

### 2.2. Inclusion and Exclusion Criteria

The present study included HF patients with an ejection fraction of <50%, namely HF with reduced ejection fraction (HFrEF) and HF with mid-range ejection fraction (HFmrEF). HF patients presenting with acute coronary syndrome and those without information regarding COPD, clinical endpoints, and beta-blockers were excluded.

### 2.3. Definitions

COPD was diagnosed based on medical records or on the clinical judgment of each investigator, taking into account the patient’s medical history, treatment, and/or spirometric data [20]. Cachexia was defined as the combination of body mass index (BMI) <20 kg/m^2^ and at least one of the following biochemical abnormalities: CRP >5 mg/L, hemoglobin <120 g/L, and/or albumin <32 g/L, according to previous studies [21,22]. To assess the impact of beta-blocker doses on prognosis, the titer of carvedilol to bisoprolol was regarded as 1:5 [23] and the higher dose group was defined as patients who took bisoprolol of ≥2.5 mg or carvedilol of ≥12.5 mg. Those who received bisoprolol or carvedilol of less than the doses were classified as the lower dose group.

### 2.4. Other Variables

Patient characteristics (including age, sex, BMI, hypertension, diabetes mellitus, dyslipidemia, atrial fibrillation/flutter (AF/AFL), COPD, history of stroke and transient ischemic attack, vital signs, New York Heart Association (NYHA) classification, an etiology of HF, left ventricular ejection fraction (LVEF), laboratory data, medication, and device therapy) and 2-year all-cause mortality were collected by cardiologists and well-trained clinical researchers. LVEF was assessed by using Simpson’s biplane method [24] during the index hospitalization after the stabilization of HF symptoms. Etiologies were classified into ischemic heart disease, diastolic cardiomyopathy, valvular heart disease, and others, all of which were determined by study committee members. Information regarding oral agents for HF such as beta-blockers, diuretics, renin-angiotensin system (RAS) inhibitors including angiotensin-converting enzyme inhibitors and angiotensin II receptor blockers, and mineralocorticoid receptor antagonists (MRA) was gathered at the time of discharge. Initial prescription of beta-blockers was introduced when acute HF was compensated. Angiotensin receptor-neprilysin inhibitors and hyperpolarization-activated cyclic nucleotide-gated ion channel inhibitors were not approved for clinical use in Japan during the study period.

### 2.5. Study Endpoints

The primary endpoint was 2-year all-cause mortality. Cardiac and noncardiac mortality were evaluated as the secondary endpoints. All mortality was reviewed and classified into cardiac- or noncardiac mortality referring to medical records. Central committee members reviewed the abstracted records and confirmed the mode of death based on the 2014 American College of Cardiology/American Heart Association key data elements and definitions for cardiovascular endpoint events in clinical trials [25].

### 2.6. Data Robustness

The data were entered into an electronic data-capturing system, which provided a robust data query engine and system validations for data quality. Exclusive on-site auditing by Y.S. and S.K. ensured proper registration of each patient.

### 2.7. Statistical Analysis

Numerical data are presented as mean ± standard deviation if the data followed a normal distribution. Otherwise, data are displayed as medians with interquartile ranges. Categorical variables are expressed as absolute numbers or percentages. Continuous variables were analyzed using an unpaired Student’s *t*-test or the Mann–Whitney U test, while Fisher’s exact test or the chi-squared test was used for categorical variables. The likelihood of prescription of beta-blockers was analyzed with logistic regression analysis in those with and without COPD and expressed as odds ratio (OR), 95% confidence interval (CI), and *p* value. The cumulative incidence of 2-year all-cause, cardiac, and non-cardiac mortality was assessed using Kaplan–Meier curve analysis with a log-rank test. The risk of each mortality was assessed using Cox regression analysis and ex-pressed as HR, 95% CI, and *p* value. To evaluate possible interaction between beta-blockers and COPD, we compared models with and without the interaction term of two variables and calculated a *p* value using the likelihood ratio test. Multivariate Cox regression analysis was conducted with forward stepwise selection. Variables in which *p* < 0.10 in the univariate Cox regression analysis were selected for adjustment. Furthermore, inverse probability weighting (IPW) was used to assess the association of beta-blockers with 2-year all-cause mortality. The probability of receiving treatment was estimated by a logistic model in which the covariables included age, sex, history of AF/AFL, chronic kidney disease (CKD) stage G3b or higher, NYHA classification, RAS inhibitors, and MRA. The outcome model was constructed using weighted means. Survival time analysis was conducted based on the Weibull model, which can accurately model the time-to-failure of real-world events. Clinical variables used to predict censoring in the censoring model contained age, sex, BMI, hypertension, dyslipidemia, diabetes mellitus, history of AF/AFL, CKD G3b or higher, NYHA classification, LVEF, RAS inhibitors, and MRA. Potential outcome means (POMs) of those who did not take beta-blockers were estimated in the process of treatment assignment. The average treatment effect (ATE), which is the absolute difference in POMs, was finally estimated. ATE and POMs were expressed as absolute number (days), 95% CI, and *p* value. Statistical significance was defined as *p* < 0.05. All statistical analyses were carried out using Stata version 14 (Stata Corp; College Station, TX, USA).

## 3. Results

### 3.1. Patient Characteristics

Of the 3634 patients, 1542 (42.4%), 230 (6.3%), 16 (0.4%), and three patients (0.1%) with LVEF ≥50%, missing data of the primary endpoint, COPD, or beta-blockers were excluded. Eventually, 1843 patients with EF <50% were included (patients with COPD, 83 [4.5%]; patients without COPD, 1760 [95.5%]). The dose of carvedilol and bisoprolol were 5 mg (2.5–10) mg (patients with COPD, 7.5 mg [2.5–10] mg; patients without COPD, 5 mg [2.5–10] mg) and 2.5 mg (1.25–2.5) mg (patients with COPD, 1.25 mg [1.25–2.5] mg; patients without COPD, 2.5 mg [1.25–2.5] mg). Patient characteristics are shown in Table 1. Cachexia was identified in 376 patients (20%) (patients with COPD, 19 [23%]; patients without COPD, 357 [20%]). There was no significant difference in background, comorbidities except for cachexia, laboratory data, medication, or device therapy between patients with COPD who took beta-blockers and those who did not. Patients without COPD who took beta-blockers presented with younger age, higher BMI, lower prevalence of NYHA classification at discharge ≥III and cachexia, higher albumin, and higher prevalence of RAS inhibitors compared to those who did not.

### 3.2. Predictors for Prescription of Beta-Blockers

The variables associated with the prescription of beta-blockers are displayed in Table 2. COPD was not correlated with the prescription of the agents. Concomitant cachexia was significantly related to a lower prescription of beta-blockers in patients with COPD.

### 3.3. Long-Term Outcomes and Clinical Impact of COPD in All the Patients

The follow-up duration was 709 (316–730) days. All-cause, cardiac, and non-cardiac mortality at two years are shown in Table 3. Notably, the incidence of non-cardiac mortality was higher in patients with COPD than in those without (*p* = 0.003), while that of cardiac mortality was similar in both groups (*p* = 0.770). COPD was associated with a higher incidence of all-cause or non-cardiac mortality (HR for all-cause mortality, 1.58; 95% CI, 1.02–2.46; *p* = 0.042; HR for non-cardiac mortality, 2.20; 95% CI, 1.29–3.74; *p* = 0.004); however, the morbidity was not related to cardiac mortality (HR for cardiac mortality, 0.93; 95% CI, 0.41–2.10; *p* = 0.861).

### 3.4. Different Impact of Beta-Blockers between Patients with and without COPD

**Figure 1:**
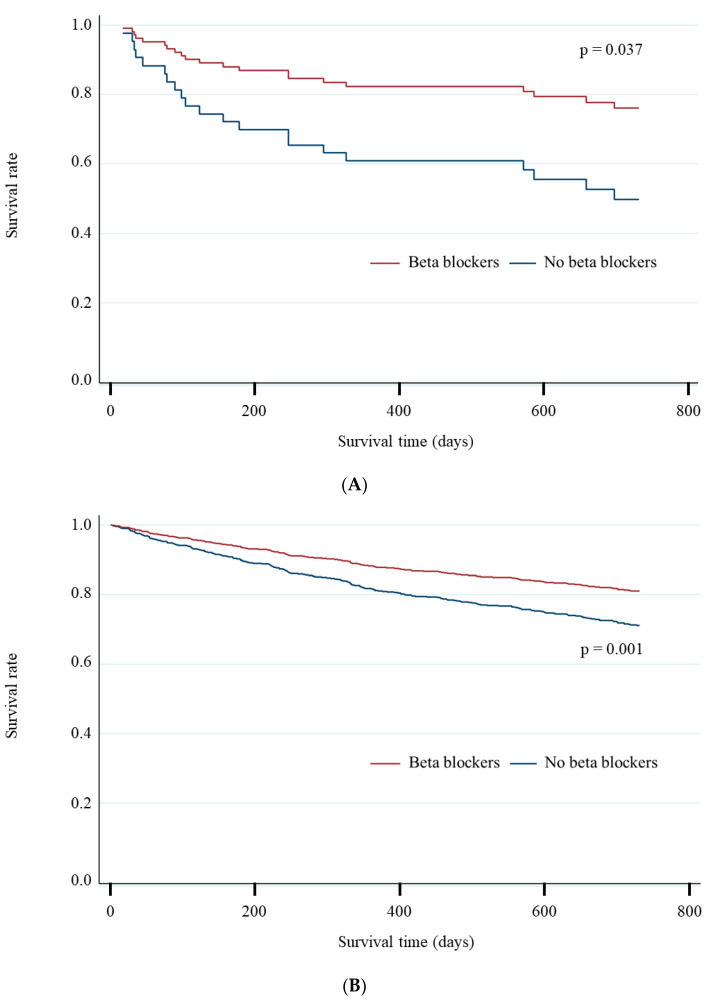
Association of beta-blockers with two-year mortality in patients with and without COPD. (**A**) Association of beta-blockers with two-year all-cause mortality in patients with COPD. (**B**) Association of beta-blockers with two-year all-cause mortality in patients without COPD. Prescription of beta-blockers was associated with a lower all-cause mortality at two years regardless of COPD (**A** and **B**).

Kaplan–Meier curve analysis indicated the association of beta-blockers with 2-year all-cause mortality in patients with and without COPD (Figure 1A, B). Table 3 demonstrates an association of beta-blockers with 2-year all-cause, cardiac, and non-cardiac mortality. Univariate Cox regression analysis indicated that beta-blockers were associated with the lower incidence of 2-year mortality in patients with and without COPD (*p* for interaction = 0.287); the association in COPD or non-COPD was due to the lower incidence of non-cardiac or cardiac mortality. The results of Cox regression analysis for all-cause mortality are displayed in Table 4. Multivariate analysis demonstrated that beta-blockers were associated with the lower incidence of 2-year all-cause mortality in patients with COPD; however, this was not observed in those without COPD. Among individuals with LVEF <40%, beta-blockers were related to the incidence of the primary endpoint regardless of the presence of concomitant COPD (adjusted HR in those with COPD, 0.57; 95% CI, 0.40–0.82; *p* = 0.003; adjusted HR in those without COPD, 0.63; 95% CI, 0.44–0.91; *p* = 0.013). Such favorable association was disappeared in the setting of cachexia irrespective of COPD (unadjusted HR in cachexic patients with COPD, 0.43; 95% CI, 0.11–1.76; *p* = 0.241; unadjusted HR in cachexic patients without COPD, 0.81; 95% CI, 0.50–1.31; *p* = 0.387). Finally, IPW demonstrated that beta-blockers extended survival time (ATE, 223 days; 95% CI, 70–375 days; *p* = 0.004), while the POM was 148 days (95% CI, 83–212 days) in those with COPD.

### 3.5. Impact of Types and Doses of Beta-Blockers on Prognosis

Compared to carvedilol, bisoprolol tended to be associated with lower 2-year all-cause mortality (HR, 0.35; 95% CI, 0.11–1.11; *p* = 0.076) and noncardiac mortality (HR, 0.22; 95% CI, 0.05–1.02; *p* = 0.053) in patients with COPD. However, there was no significant difference in cardiac mortality between the agents (HR, 0.89; 95% CI, 0.12–6.33; *p* = 0.906). Different impacts of such agents on any endpoints were not observed in patients without COPD. The higher and lower dose groups included 340 and 1198 patients, respectively. The higher dose group was associated with lower 2-year all-cause mortality in patients without COPD (HR, 0.69; 95% CI, 0.49–0.97; *p* = 0.033), but was not in those with COPD (HR, 1.34; 95% CI, 0.45–4.02; *p* = 0.600) in comparison to the lower dose group. The favorable association in patients without COPD was due to lower cardiac mortality (HR, 0.56; 95% CI, 0.33–0.97; *p* = 0.038). Doses of the agents were not related to 2-year cardiac mortality (HR, 2.22; 95% CI, 0.31–15.88; *p* = 0.427) or noncardiac mortality (HR, 1.07; 95% CI, 0.28–4.16; *p* = 0.920) in those with COPD.

## 4. Discussion

The present study demonstrated that beta-blockers were prescribed frequently in Japanese clinical practice and were correlated with better prognosis even in HF patients with COPD; however, the result was due to the reduced non-cardiac mortality, not cardiac mortality. Beta-blockers were prescribed less frequently for patients with cachexia, which could be due to HF and/or COPD. These findings imply that beta-blockers might be prescribed for patients who were regarded as not having poor prognosis by clinicians.

While previous studies have suggested that patients with multiple comorbidities often had low efficacies of treatments confirmed by randomized controlled studies or expected by cohort studies [26,27,28,29,30], the impact of beta-blockers on all-cause mortality did not seem to be attenuated in patients with COPD at a glance. However, such results might be derived from selection biases considering the reduced non-cardiac, non-affected cardiac mortality, and a lower prevalence of cachexia in COPD patients with beta-blockers. We should recognize the potential selection bias and interpret these results carefully. In contrast, the lower incidence of all-cause mortality was due to the lower incidence of cardiac mortality in those without COPD. Similar potential biases were observed in the different impacts of beta-blockers among the cohort studies [7,31,32] and a randomized controlled study [9]. It has remained unknown whether the advantages of beta-blockers might balance or be inferior to the disadvantages in HF patients with COPD who take beta-blockers. While these agents provide benefits for HF patients through the suppression of harmful neurohormonal changes evoked by HF [33], which is also activated and in the setting of COPD [34], they might theoretically cause negative effects on airflow. In fact, the BLOCK-COPD trial demonstrated the potential harm of beta-blockers [9]. A previous report indicated that cachexia was associated with COPD severity [35]. Considering the lack of association between the agents and endpoints in cachexic COPD patients, it may be difficult to expect the merit of beta-blockers in the setting of severe COPD.

A possible interaction between HF and COPD should also be noted. The chronic inflammation due to COPD may worsen the functional status and hemodynamics of HF patients. The underuse and underdosing of beta-blockers may contribute to exacerbation of HF, while up-titration of the agents may exacerbate COPD [9]. Net clinical benefit of beta-blockers in HF patients with COPD should be investigated in the future.

It is noteworthy that the present study included a very high prescription rate of beta-blockers, as high as more than 80%, which is higher than that reported in previous studies [3,4,20]. It would also be noteworthy that the dose was lower than the recommended doses [1]; however, the sensitivity for beta-blockers differs among races and is higher in Asians than in Caucasians [15,16].

Evaluation of concomitant non-cardiac comorbidities becomes increasingly important in an aging society, in which more than two-thirds who develop cardiovascular diseases present with non-cardiovascular comorbidities [36,37]. Contemporary clinical practice and research have mainly focused on single diseases that do not include complexities imposed by concurrent comorbidities [38]. Therefore, further studies are needed to investigate treatment benefits of guideline-directed medical therapy on HF with non-cardiac comorbidities such as in the present study. The clinical impact of beta-blockers based on COPD severity should be evaluated in the future because causes of mortality differed based on severity [39]. The main causes of death in mild or moderate COPD are lung cancer and cardiovascular diseases, but the predominant cause in severe COPD is respiratory failure. It is plausible that the efficacy of beta-blockers would not be significant in patients with more severe COPD. The classification of COPD severity may contribute to our understanding of the association of beta-blockers with prognosis in HF patients with COPD. The underestimation of COPD has also been regarded as a problem in HF patients with COPD [40]. Furthermore, appropriate titration of beta-blockers in the setting of HF and COPD remains unclear. Underdosing may have led to a lack of association between beta-blockers and cardiac mortality. The dosage of beta-blockers has recently been chosen as new performance measure for HF to address the frequent lack of its titration. A previous study demonstrated that most eligible HFrEF patients did not receive target doses of medical therapy at any point during follow-up, and few patients had their doses increased over time [40]. Such clinical inertia should be recognized and must be resolved. Therefore, future studies evaluating HF and COPD should perform a pulmonary function test and pursue up/down-titration or discontinuation of beta-blockers after discharge.

### Limitations

Our study has several limitations. First, there might have been an underestimation of COPD because a respiratory function test was not performed on all hospitalized patients. Indeed, the prevalence of this morbidity was much lower than in Western countries. The prevalence of COPD ranges from 11% to 52% in North America and from 9% to 41% in Europe [13]. However, the prevalence may differ based on race. Previous reports have indicated that the prevalence of COPD is lower in Asia, where it ranged from 5% to 8% [5,14]. Considering these findings, the prevalence of COPD patients in our study might be appropriate. Second, the lack of a pulmonary function test and detailed data regarding COPD (e.g., duration of COPD, long-term oxygen supply) are also limitations in terms of evaluating COPD severity. Third, the number of COPD patients was small, and the results of multivariate Cox regression analysis were not necessarily robust due to the sample size. However, IPW would reinforce against any potential vulnerability. Finally, the present study had some biases including the aforementioned selection bias and immortal time bias. Beta-blockers were not prescribed in the manner of a blinded randomized controlled trial. Therefore, the association of beta-blockers on mortality should be interpreted carefully in cohort studies.

## 5. Conclusions

The prescription of beta-blockers was associated with lower all-cause mortality in HF patients with LVEF <50% regardless of COPD; however, the results were derived from lower non-cardiac mortality in those with COPD and lower cardiac mortality in those without. These findings may reflect selection biases in the prescription of beta-blockers in the setting of COPD. The actual situation of the prescription of beta-blockers after discharge such as withdrawal and up-titration should be clarified in the future. Furthermore, randomized controlled studies based on COPD severity are necessary to determine the clinical significance of these agents in such patients.

## Figures and Tables

**Table 1:** Patient characteristics.

	All	Patients with COPD				Patients without COPD			
		All	Beta-Blocker	No Beta- Blocker	*p* Value	All	Beta-Blocker	No Beta- Blocker	*p* Value
	*n* = 1843	*n* = 83	*n* = 67	*n* = 16		*n* = 1760	*n* = 1509	*n* = 251	
Age, years	72 ± 14	75 ± 11	75 ± 10	75 ± 15	0.974	71 ± 14	71 ± 14	77 ± 14	<0.001
Male, *n* (%)	1271 (69)	72 (87)	58 (87)	14 (88)	1.000	1199 (68)	1034 (69)	165 (66)	0.381
BMI, kg/m^2^	22 ± 4	21 ± 4	22 ± 4	20 ± 4	0.186	22 ± 4	22 ± 4	21 ± 4	0.004
Hypertension, *n* (%)	1224 (67)	57 (70)	46 (69)	11 (69)	1.000	1167 (67)	1006 (67)	161 (64)	0.448
Dyslipidemia, *n* (%)	760 (42)	32 (40)	28 (43)	4 (25)	0.257	728 (42)	642 (43)	86 (34)	0.011
Diabetes mellitus, *n* (%)	686 (37)	23 (28)	21 (31)	2 (13)	0.213	663 (38)	580 (38)	83 (33)	0.101
Atrial fibrillation, *n* (%)	778 (42)	34 (41)	28 (42)	6 (38)	1.000	744 (42)	645 (43)	99 (39)	0.315
Previous heart failure admission, *n* (%)	582 (32)	31 (37)	27 (40)	4 (25)	0.389	551 (32)	467 (31)	84 (33)	0.440
History of ischemic stroke, *n* (%)	233 (13)	7 (8)	6 (9)	1 (6)	1.000	226 (13)	182 (12)	44 (18)	0.016
Hemodialysis, *n* (%)	60 (3)	2 (2)	1 (1)	1 (6)	0.350	58 (3)	48 (3)	10 (4)	0.565
Etiologies of heart failure									
Ischemic heart disease, *n* (%)	688 (37)	30 (36)	27 (40)	3 (19)	0.107	658 (37)	574 (38)	84 (33)	0.166
Diastolic cardiomyopathy, *n* (%)	432 (23)	21 (25)	17 (25)	4 (25)	0.975	411 (23)	378 (25)	33 (13)	<0.001
Valvular heart disease, *n* (%)	266 (14)	10 (12)	5 (7)	5 (31)	0.009	256 (15)	198 (13)	58 (23)	<0.001
Others, *n* (%)	457 (25)	22 (27)	18 (27)	4 (25)	0.879	435 (25)	359 (24)	76 (30)	0.027
NYHA classification at discharge ≥III, *n* (%)	374 (20)	21 (25)	15 (22)	6 (38)	0.218	353 (20)	278 (18)	75 (30)	<0.001
Cachexia, *n* (%)	376 (20)	19 (23)	12 (18)	7 (44)	0.044	357 (20)	287 (19)	70 (28)	0.001
Laboratory data at discharge									
Sodium, mmol/L	138 ± 3	138 ± 4	138 ± 3	137 ± 6	0.519	139 ± 3	139 ± 3	138 ± 4	0.042
Potassium, mmol/L	4.4 ± 0.5	4.4 ± 0.5	4.4 ± 0.5	4.3 ± 0.5	0.391	4.4 ± 0.5	4.4 ± 0.5	4.3 ± 0.5	0.041
Creatinine, mg/dl	1.1 (0.8–1.5)	1.2 (0.9–1.5)	1.2 (0.9–1.5)	1.0 (0.9–1.6)	0.695	1.1 (0.8–1.4)	1.1 (0.8–1.4)	1.1 (0.9–1.5)	0.519
eGFR, ml/min/m^2^	50 (35–64)	44 (36–59)	44 (36–60)	54 (34–58)	0.890	50 (35–64)	51 (35–65)	46 (32–62)	0.047
Hemoglobin, g/L	12.4 ± 2.3	12.1 ± 2.0	12.2 ± 1.9	11.5 ± 2.0	0.151	12.5 ± 2.3	12.6 ± 2.3	11.8 ± 2.2	<0.001
Albumin, g/L	3.5 ± 0.6	3.4 ± 0.5	3.4 ± 0.5	3.4 ± 0.3	0.985	3.5 ± 0.6	3.5 ± 0.5	3.4 ± 0.6	<0.001
Echocardiography									
LVEF, %	34 ± 9	33 ± 9	33 ± 9	33 ± 9	0.949	34 ± 9	33 ± 9	36 ± 9	<0.001
Medication at discharge									
Beta-blockers, *n* (%)	1576 (86)	67 (81)	67 (100)	0 (0)	<0.001	1509 (86)	1509 (100)	0 (0)	<0.001
Carvedilol, *n* (%)	1143 (62)	34 (41)				1109 (63)			
Bisoprolol, *n* (%)	415 (23)	32 (39)				383 (22)			
The others, *n* (%)	18 (1)	1 (1)				17 (1)			
RAS inhibitors, *n* (%)	1253 (68)	55 (66)	47 (70)	8 (50)	0.148	1198 (68)	1063 (70)	135 (54)	<0.001
MRA, *n* (%)	806 (44)	28 (34)	25 (37)	3 (19)	0.240	778 (44)	672 (45)	106 (42)	0.518
Furosemide, *n* (%)	1401 (76)	66 (80)	53 (79)	13 (81)	1.000	1335 (76)	1156 (77)	179 (72)	0.083
Tolvaptan, *n* (%)	72 (5)	3 (4)	2 (3)	1 (8)	0.436	69 (5)	59 (5)	10 (4)	1.000
Statin, *n* (%)	712 (39)	33 (40)	28 (42)	5 (31)	0.573	679 (39)	612 (41)	67 (27)	<0.001
Devices at discharge									
Pacemaker implantation, *n* (%)	167 (9)	6 (7)	5 (7)	1 (7)	1.000	161 (9)	120 (8)	41 (16)	<0.001
ICD, *n* (%)	136 (7)	6 (7)	5 (7)	1 (7)	1.000	130 (7)	114 (8)	16 (6)	0.602
CRT, *n* (%)	73 (4)	2 (2)	2 (3)	0 (0)	1.000	71 (4)	66 (4)	5 (2)	0.083

BMI, body mass index; CI, confidence interval; COPD, chronic obstructive pulmonary disease; CRT, cardiac resynchronization therapy; eGFR, estimated glomerular filtration rate; HR, hazard ratio; ICD, implantable cardioverter defibrillator; LVEF, left ventricular ejection fraction; MRA, mineralocorticoid receptor antagonists; NA, not applicable; NYHA, New York Heart Association; RAS, renin-angiotensin system.

**Table 2:** Contributors for the prescription of beta-blockers.

	All						Patients with COPD		
	Univariate			Multivariate			Univariate		
	OR	95% CI	*p* Value	OR	95% CI	*p* Value	OR	95% CI	*p* Value
Age (an increase of 1 year)	0.97	0.96–0.98	<0.001	0.98	0.97–0.99	<0.001	1.00	0.95–1.05	0.974
Male	1.11	0.84–1.46	0.463	NA			0.92	0.18–4.74	0.921
BMI (an increase of 1 kg/m^2^)	1.06	1.02–1.10	0.002	NA			1.10	0.95–1.28	0.187
Hypertension	1.11	0.84–1.45	0.462	NA			1.05	0.32–3.40	0.941
Dyslipidemia	1.48	1.12–1.94	0.005	NA			2.27	0.66–7.79	0.193
Diabetes mellitus	1.32	1.00–1.74	0.048	NA			3.20	0.67–15.34	0.147
COPD	0.70	0.40–1.22	0.207	NA			NA		
Atrial fibrillation	1.15	0.89–1.50	0.290	NA			1.20	0.39–3.68	0.754
Previous heart failure admission	0.93	0.71–1.23	0.619	NA			2.03	0.59–6.94	0.262
History of ischemic stroke	0.67	0.47–0.95	0.025	0.63	0.43–0.92	0.016	1.48	0.16–13.20	0.728
Hemodialysis	0.75	0.38–1.46	0.393	NA			0.23	0.01–3.84	0.304
Ischemic heart disease	1.28	0.97–1.68	0.083	NA			2.93	0.76–11.25	0.118
NYHA classification at discharge ≥III	0.52	0.39–0.70	<0.001	0.71	0.52–0.98	0.037	0.48	0.15–1.54	0.217
Cachexia	0.58	0.43–0.77	<0.001				0.28	0.09–0.90	0.033
Laboratory data at discharge									
Sodium (an increase of 1 mmol/L)	1.04	1.00–1.08	0.030	NA			1.05	0.91–1.20	0.515
Potassium (an increase of 1 mmol/L)	1.35	1.03–1.76	0.031	NA			1.66	0.53–5.24	0.387
Creatinine (an increase of 20 μmol/L)	0.99	0.97–1.01	0.378	NA			1.01	0.87–1.17	0.886
eGFR (an increase of 10 mL/min/m^2)^	1.00	0.95–1.05	0.928	NA			1.04	0.82–1.32	0.744
Hemoglobin (an increase of 10 g/L)	1.18	1.11–1.25	<0.001	NA			1.24	0.91–1.68	0.169
Albumin (an increase of 10 g/L)	1.78	1.39–2.26	<0.001	1.35	1.04–1.76	0.025	0.99	0.29–3.32	0.985
Echocardiography									
LVEF (an absolute increase of 10%)	0.73	0.63–0.85	<0.001	0.78	0.66–0.91	0.002	0.98	0.54–1.78	0.948
Medication at discharge									
RAS inhibitors	2.07	1.59–2.69	<0.001	2.02	1.52–2.68	<0.001	2.35	0.77–7.14	0.132
MRA	1.14	0.88–1.49	0.315	NA			2.58	0.67–9.94	0.169
Furosemide	1.27	0.95–1.71	0.106	NA			0.87	0.22–3.50	0.849
Tolvaptan	0.96	0.50–1.85	0.901	NA			0.39	0.03–4.71	0.461
Statin	1.85	1.39–2.47	<0.001	2.02	1.48–2.76	<0.001	1.58	0.49–5.05	0.441
Devices at discharge									
Pacemaker implantation	0.46	0.32–0.67	<0.001	NA			1.13	0.12–10.44	0.915
ICD	1.20	0.71–2.03	0.501	NA			1.13	0.12–10.44	0.915
CRT	2.36	0.94–5.90	0.067	NA			NA		

BMI, body mass index; CI, confidence interval; COPD, chronic obstructive pulmonary disease; CRT, cardiac resynchronization therapy; eGFR, estimated glomerular filtration rate; ICD, implantable cardioverter defibrillator; LVEF, left ventricular ejection fraction; MRA, mineralocorticoid receptor antagonists; NA, not applicable; NYHA, New York Heart Association; OR, odds ratio; RAS, renin-angiotensin system.

**Table 3:** An association of beta-blockers with 2-year prognosis in patients with and without COPD.

	All	Patients with COPD					Patients without COPD				*p* Value
		All	Beta- Blockers	No Beta-Blockers	HR (95% CI)	*p* Value	All	Beta- Blockers	No Beta-Blockers	HR (95% CI)	
	(*n* = 1843)	(*n* = 83)	(*n* = 67)	(*n* = 16)			(*n* = 1760)	(*n* = 1509)	(*n* = 251)		
All-cause mortality, *n* (%)	315 (17)	21 (25)	14 (21)	7 (44)	0.39 (0.16–0.98)	0.044	294 (17)	238 (16)	56 (22)	0.62 (0.46–0.83)	0.001
Cardiac mortality, *n* (%)	149 (8)	6 (7)	4 (6)	2 (13)	0.37 (0.07–2.01)	0.248	143 (8)	108 (7)	35 (14)	0.45 (0.31–0.66)	<0.001
Noncardiac mortality, *n* (%)	166 (9)	15 (18)	10 (15)	5 (31)	0.40 (0.14–1.18)	0.098	151 (9)	130 (9)	21 (8)	0.90 (0.57–1.42)	0.647

CI, confidence interval; COPD, chronic obstructive pulmonary disease; HR, hazard ratio.

**Table 4:** Cox regression analysis for all-cause mortality at two years.

	Patients with COPD						Patients without COPD					
	Univariate			Multivariate			Univariate			Multivariate		
	HR	95% CI	*p* Value	HR	95% CI	*p* Value	HR	95% CI	*p* Value	HR	95% CI	*p* Value
Age (an increase of 1 year)	1.07	1.01–1.12	0.017	NA			1.05	1.04–1.06	<0.001	1.04	1.02–1.05	<0.001
Male	0.65	0.22–1.93	0.435	NA			0.85	0.67–1.08	0.195	NA		
BMI (an increase of 1 kg/m^2^)	0.96	0.86–1.07	0.467	NA			0.88	0.85–0.91	<0.001	NA		
Hypertension	0.47	0.20–1.11	0.086	0.33	0.13–0.85	0.021	1.03	0.81–1.32	0.791	NA		
Dyslipidemia	0.32	0.11–0.94	0.039	NA			1.12	0.89–1.41	0.334	NA		
Diabetes mellitus	0.41	0.12–1.41	0.158	NA			1.26	1.00–1.58	0.053	NA		
Atrial fibrillation	0.53	0.20–1.36	0.184	NA			1.03	0.81–1.29	0.821	NA		
Previous heart failure admission	0.70	0.28–1.73	0.437	NA			1.75	1.39–2.21	<0.001	NA		
History of ischemic stroke	0.48	0.06–3.58	0.474	NA			1.28	0.93–1.76	0.129	NA		
Hemodialysis	1.87	0.25–13.94	0.541	NA			3.01	1.93–4.69	<0.001	1.86	1.09–3.18	0.023
Ischemic heart failure	0.71	0.27–1.82	0.472	NA			1.50	1.20–1.89	<0.001	NA		
NYHA classification at discharge ≥III	3.72	1.57–8.80	0.003	NA			2.56	2.02–3.25	<0.001	1.80	1.39–2.33	<0.001
Cachexia	2.19	0.91–5.28	0.082	NA			2.67	2.10–3.39	<0.001	NA		
Laboratory data at discharge												
Sodium (an increase of 1 mmol/L)	0.90	0.81–0.99	0.038	NA			0.92	0.89–0.94	<0.001	0.95	0.92–0.98	<0.001
Potassium (an increase of 1 mmol/L)	0.63	0.25–1.57	0.320	NA			0.91	0.72–1.16	0.448	NA		
Creatinine (an increase of 20 μmol/L)	1.01	0.90–1.12	0.921	NA			1.03	1.02–1.04	<0.001	NA		
eGFR (an increase of 10 mL/min/m^2)^	1.21	0.99–1.48	0.058	NA			0.78	0.74–0.83	<0.001	0.92	0.86–0.98	0.007
Hemoglobin (an increase of 10 g/L)	0.74	0.57–0.95	0.020	NA			0.75	0.71–0.79	<0.001	0.88	0.81–0.95	0.002
Albumin (an increase of 10 g/L)	0.27	0.09–0.78	0.015	0.22	0.08-0.63	0.005	0.38	0.31–0.46	<0.001	0.55	0.42–0.70	<0.001
Echocardiography												
LVEF (an absolute increase of 10%)	1.19	0.71–1.99	0.503	NA			0.87	0.77–0.98	0.020	0.68	0.59–0.79	<0.001
Medication at discharge												
Beta-blockers	0.39	0.16–0.98	0.044	0.36	0.14-0.92	0.033	0.62	0.46–0.83	0.001	NA		
RAS inhibitors	0.63	0.26–1.49	0.289	NA			0.51	0.40–0.64	<0.001	0.74	0.57–0.96	0.024
MRA	0.78	0.30–2.02	0.616	NA			0.86	0.68–1.08	0.189	NA		
Furosemide	1.50	0.44–5.10	0.516	NA			1.23	0.93–1.63	0.150	NA		
Tolvaptan	NA			NA			2.83	1.86–4.31	<0.001	1.98	1.27–3.08	0.003
Statin	1.02	0.81–1.28	0.861	NA			1.02	0.81–1.30	0.840	NA		
Devices at discharge												
Pacemaker implantation	0.59	0.08–4.42	0.609	NA			1.30	0.92–1.85	0.135	NA		
ICD	0.61	0.08–4.57	0.631	NA			1.24	0.85–1.82	0.270	NA		
CRT	NA			NA			1.29	0.78–2.14	0.321	NA		

BMI, body mass index; CI, confidence interval; COPD, chronic obstructive pulmonary disease; CRT, cardiac resynchronization therapy; eGFR, estimated glomerular filtration rate; HR, hazard ratio; ICD, implantable cardioverter defibrillator; LVEF, left ventricular ejection fraction; MRA, mineralocorticoid receptor antagonists; NA, not applicable; NYHA, New York Heart Association; RAS, renin-angiotensin system.

## Data Availability

Data available on request due to ethical restrictions. The data presented in this study are available on request from the corresponding author. The data are not publicly available because the ethical committee in each center has not permitted data disclosure.
